# Supplementary material for: A snow-fire bridge mechanism for the 2025 Southern California winter wildfire
Source: Nat Commun. 2026 Mar 19;17:4169. doi: 10.1038/s41467-026-70827-z (PMC13153211; doi:10.1038/s41467-026-70827-z)
Supplement: Supplementary file 1 — Supplementary Information [file 41467_2026_70827_MOESM1_ESM.pdf]

Supplementary Information for

A Snow-Fire Bridge Mechanism for the 2025 Southern California Winter Wildfire

Shizuo Liu*[1], Shineng Hu*[1,2] and Richard Seager[3]

1. Division of Earth and Climate Sciences, Nicholas School of the Environment, Duke University, Durham, NC, USA
2. Department of Civil and Environmental Engineering, Duke University, Durham, NC, USA
3. Lamont Doherty Earth Observatory of Columbia University, Palisades, NY, USA

*Corresponding author: Shizuo Liu (shizuo.liu@duke.edu); Shineng Hu (shineng.hu@duke.edu)

This PDF contains 20 figures and 2 tables.

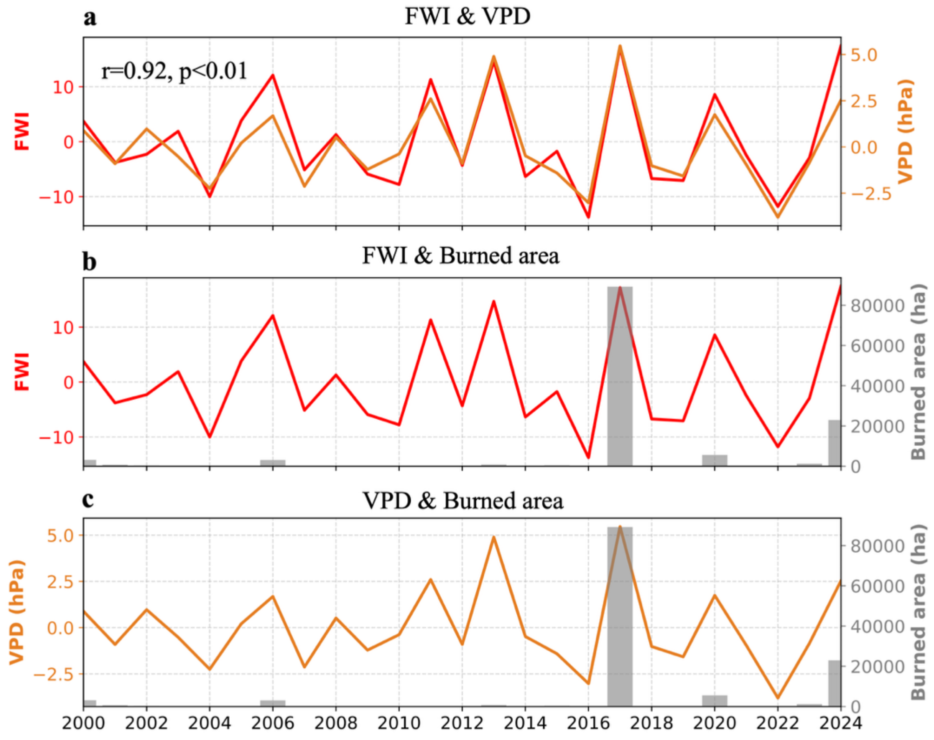

Supplementary Fig. 1: Southern California December-January fire weather index (FWI), vapor pressure deficit (VPD) and burned area. a Time series of Southern California December-January (DJ) gridMET FWI (unitless, the red line) and gridMET VPD (hPa, the orange line). **b** Time series of Southern California DJ gridMET FWI (the red line) and MTBS burned area (ha, the gray bars). **c** Time series of Southern California DJ gridMET VPD (the orange line) and MTBS burned area (the gray bars). The year of DJ is defined by the year of its December.

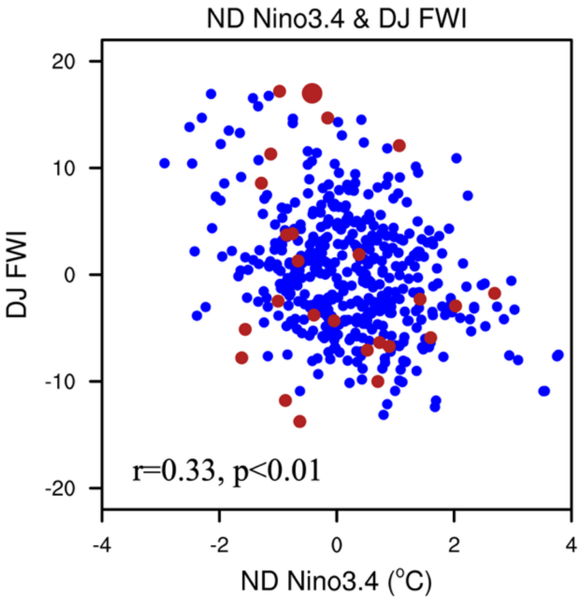

Supplementary Fig. 2: Relationship between Niño3.4 and Southern California fire weather index (FWI) in observations and CMIP6 simulations. Scatter plot showing the relationship between the November-December (ND) Niño3.4 (°C) and Southern California December-January (DJ) FWI (unitless) in observations from 2000 to 2024 (red dots) and 44 CMIP6 simulations from 2020 to 2029 (the blue dots, with a sample size of 440 and a correlation coefficient of -0.33, p<0.01). The bigger, red dot represents the observed ND Niño3.4 index in 2024 and the average Southern California gridMET FWI in December 2024-January 2025.

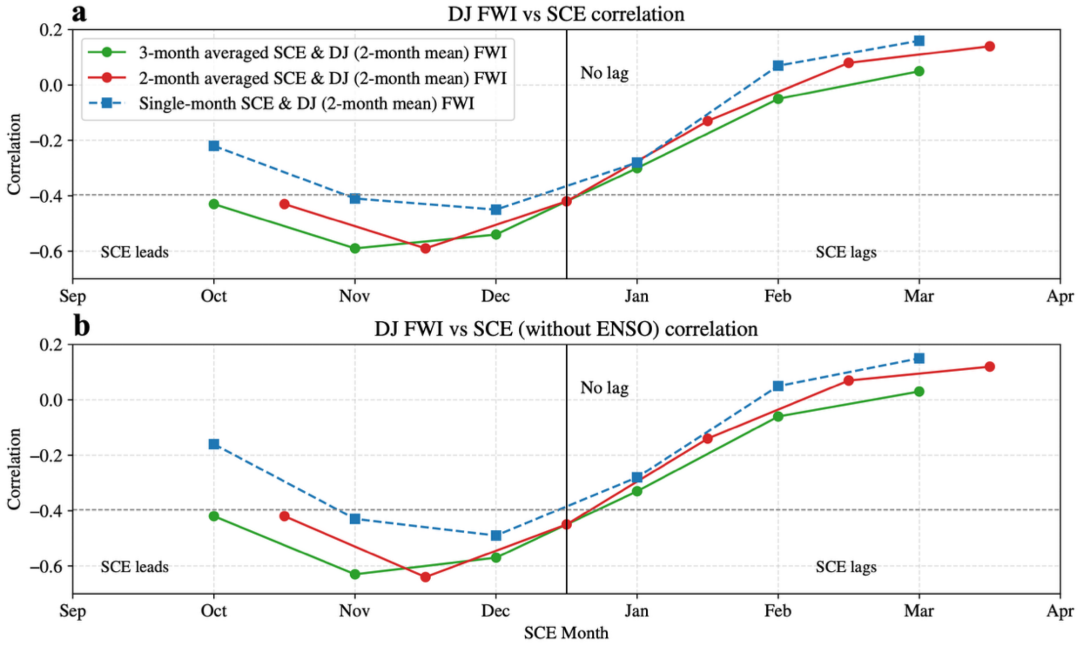

Supplementary Fig. 3: Connections between western Eurasian snow cover extent (SCE) and Southern California fire weather index (FWI). a Correlation coefficients between western Eurasian SCE during different time periods and the December-January (DJ) averaged California FWI. The green solid line shows the correlations between 3-month averaged SCE and DJ FWI, with the red solid line showing the correlations between 2-month averaged SCE and DJ FWI, and the blue dashed line showing the correlations between single-month SCE and DJ FWI. The horizontal axis represents the central time point of the SCE period. **b** Same as **a**, but for the indices with the El Niño-Southern Oscillation (ENSO) signal removed.

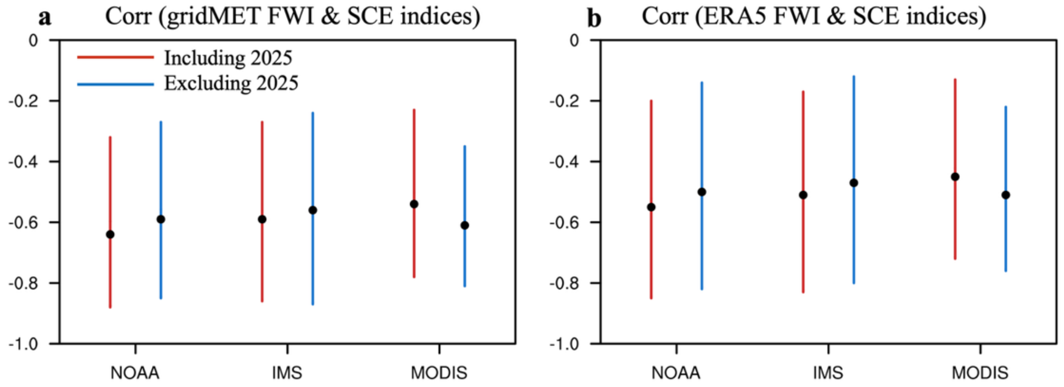

Supplementary Fig. 4: Bootstrap-based assessment of western Eurasian snow cover extent (SCE) and Southern California fire weather index (FWI) correlation coefficients. Correlation coefficients between November–December western Eurasian SCE indices and December–January Southern California FWI indices. SCE is derived from three datasets (NOAA, IMS, and MODIS), and FWI is derived from (**a**) gridMET and (**b**) ERA5, with El Niño-Southern Oscillation (ENSO) removed. Correlations are calculated both including 2025 (red; N = 25) and excluding 2025 (blue; N = 24). Black dots indicate the median correlation coefficients, and vertical bars denote the 95% confidence intervals estimated using a nonparametric bootstrap resampling approach with 10,000 iterations.

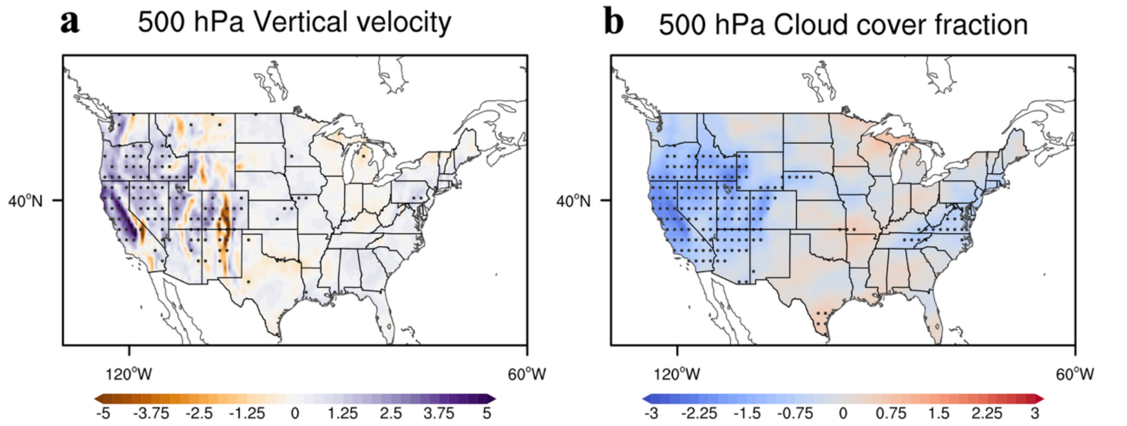

Supplementary Fig. 5: Observed vertical velocity and cloud cover fraction associated with western Eurasian snow cover extent (SCE) reduction. a, b Regression of December-January 500 hPa vertical velocity (10^{-2} Pa s^{-1}; downward positive) (**a**), and 500 hPa cloud cover fraction (%) (**b**) against normalized NOAA November-December western Eurasian SCE index (the blue dashed curve in Fig. 1a, multiplied by –1 to represent reduction) with El Niño-Southern Oscillation (ENSO) removed. Stippling in panels (**a**–**b**) indicates regions with regression coefficients significant at the 90% confidence level based on the two-sided Student's t-test.

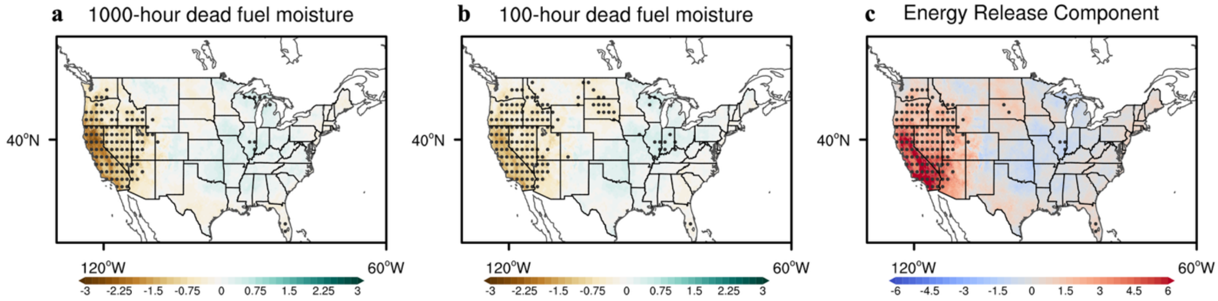

Supplementary Fig. 6: Observed dead fuel moisture and potential fire intensity associated with western Eurasian snow cover extent (SCE) reduction. Regression of December-January average (**a**) 1000-hour dead fuel moisture (%), (**b**) 100-hour dead fuel moisture (%) and (**c**) Energy Release Component (unitless) against the normalized NOAA November-December average western Eurasian SCE index (the blue dashed line in Fig. 1a, multiplied by –1 to represent reduction) with El Niño-Southern Oscillation (ENSO) removed. Stippling areas in panels (**a-c**) indicate responses significant at the 90% confidence level based on the two-sided Student's t-test.

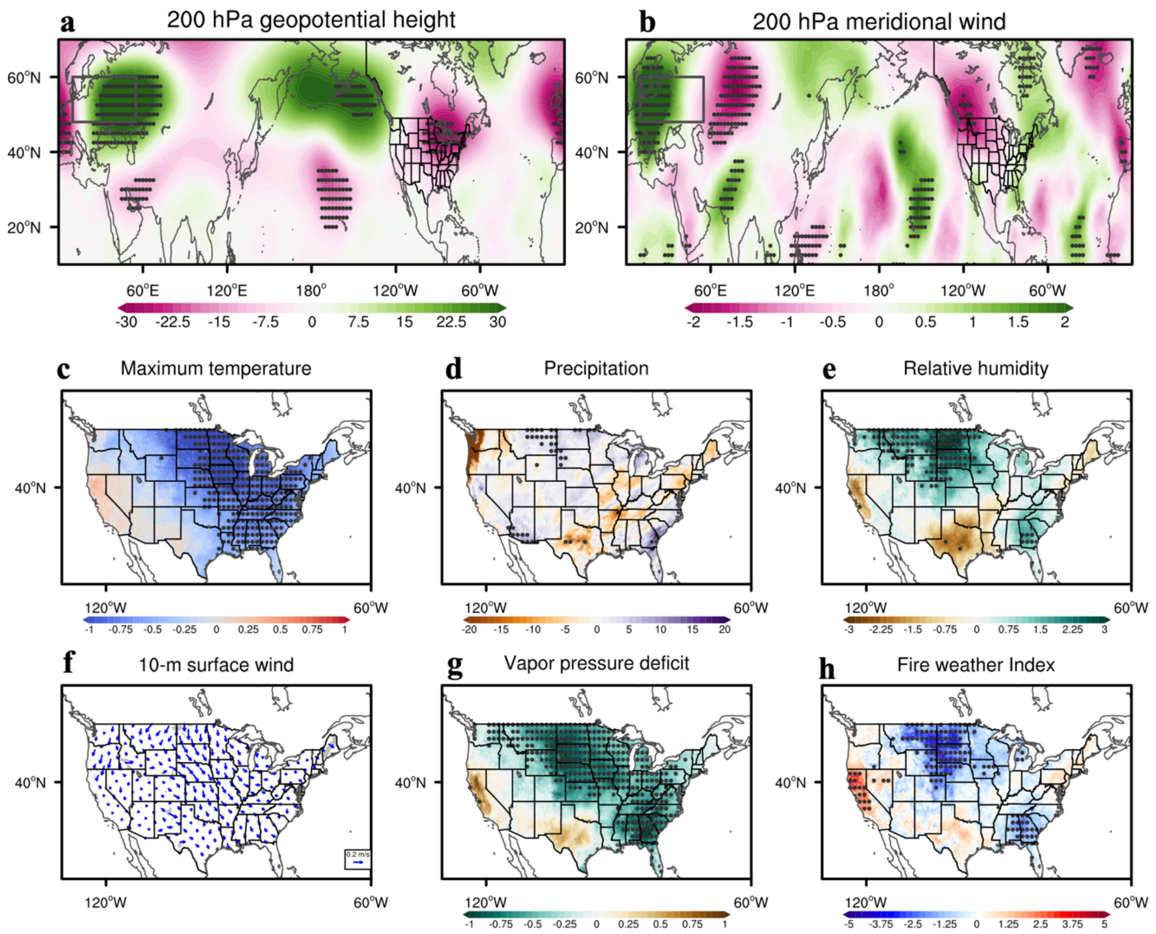

Supplementary Fig. 7: Observed November atmospheric teleconnection and U.S. fire-weather condition associated with western Eurasian snow cover extent (SCE) reduction. Linear regression of November (**a**) 200 hPa geopotential height (m), (**b**) 200 hPa meridional wind (m/s), (**c**) maximum temperature (°C), (**d**) precipitation (mm/month), (**e**) relative humidity (%), (**f**) 10 m surface wind, (**g**) vapor pressure deficit (VPD, hPa), and (**h**) fire weather index (FWI, unitless) against the normalized NOAA November western Eurasian SCE index (multiplied by –1 to represent reduction) with El Niño-Southern Oscillation (ENSO) removed. Stippling areas in panels (**a**–**e**) and (**g**-**h**), and shading areas in panel (**f**) indicate regions with regression coefficients significant at the 90% confidence level based on the two-sided Student's t-test. The black boxes in panels (**a**) and (**b**) represent the domain of western Eurasia (10 °E-55 °E & 48 °N-60 °N).

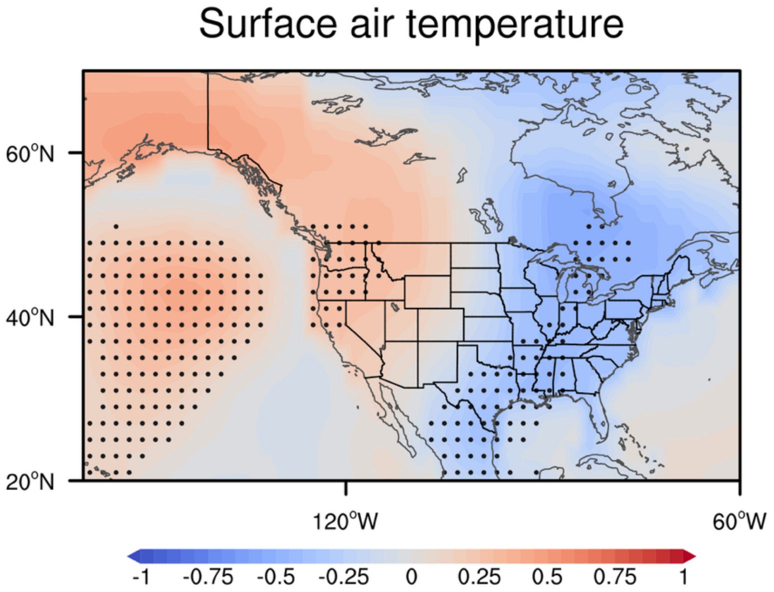

Supplementary Fig. 8: Observed surface air temperature associated with western Eurasian snow cover extent (SCE) reduction. Regression of December-January GISS surface air temperature (°C) against normalized NOAA November-December western Eurasian SCE index (the blue dashed curve in Fig. 1a, multiplied by –1 to represent reduction) with El Niño-Southern Oscillation (ENSO) removed. Stippling regions indicate regression coefficients significant at the 90% confidence level based on the two-sided Student's t-test.

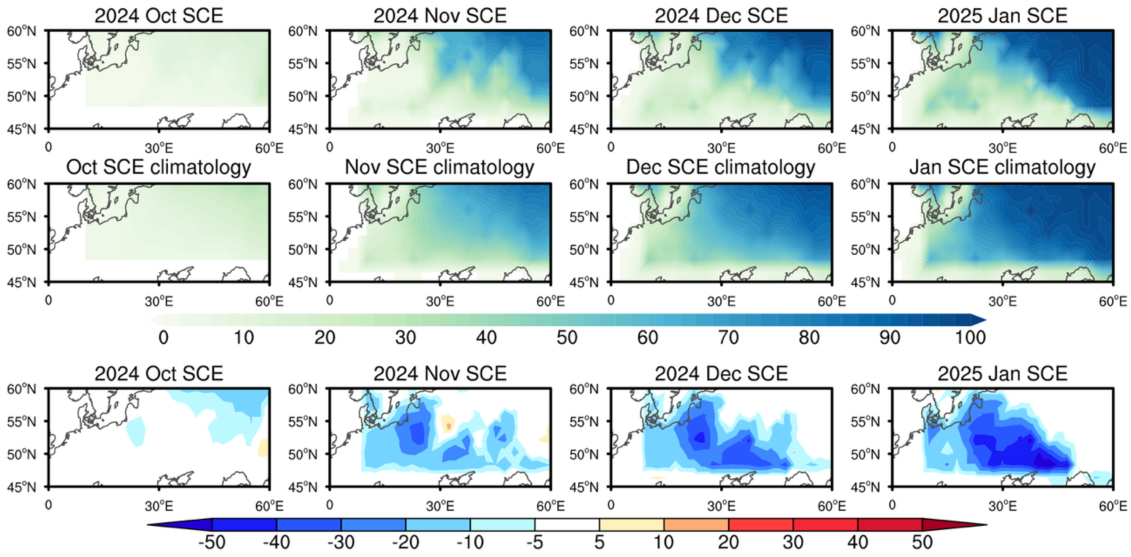

Supplementary Fig. 9: Snow cover forcing used in the fully coupled CESM2.1 experiments. Top row: NOAA snow cover extent (SCE, %) from October 2024 to January 2025. Middle row: Climatology of NOAA SCE from October to January (2000–2025). Bottom row: NOAA SCE anomalies from October 2024 to January 2025 (Data and Methods). The first two rows show the snow forcing in the LessSCE and ClimSCE experiments, respectively, while the last row shows the difference between the two (LessSCE minus ClimSCE). The SCE forcing is prescribed over western Eurasia region (10 °E-55 °E & 48 °N-60 °N).

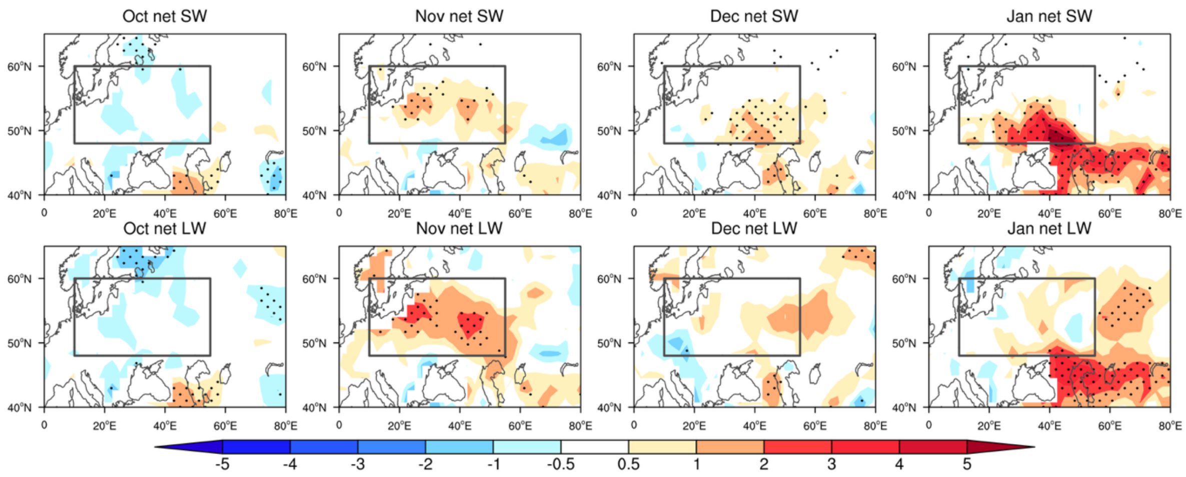

Supplementary Fig. 10: Simulated net shortwave and longwave radiative flux responses. Monthly responses of net shortwave radiative flux (positive downward, top row) and net longwave radiative flux (positive upward, bottom row). The unit is W/m^2. Each response is the ensemble mean difference between the two simulations in the experiment (LessSCE minus ClimSCE). Stippling areas indicate values significant at the 90% confidence level based on the two-sided Student's t-test. The black box in each panel represents the domain of western Eurasia (10 °E-55 °E & 48 °N-60 °N).

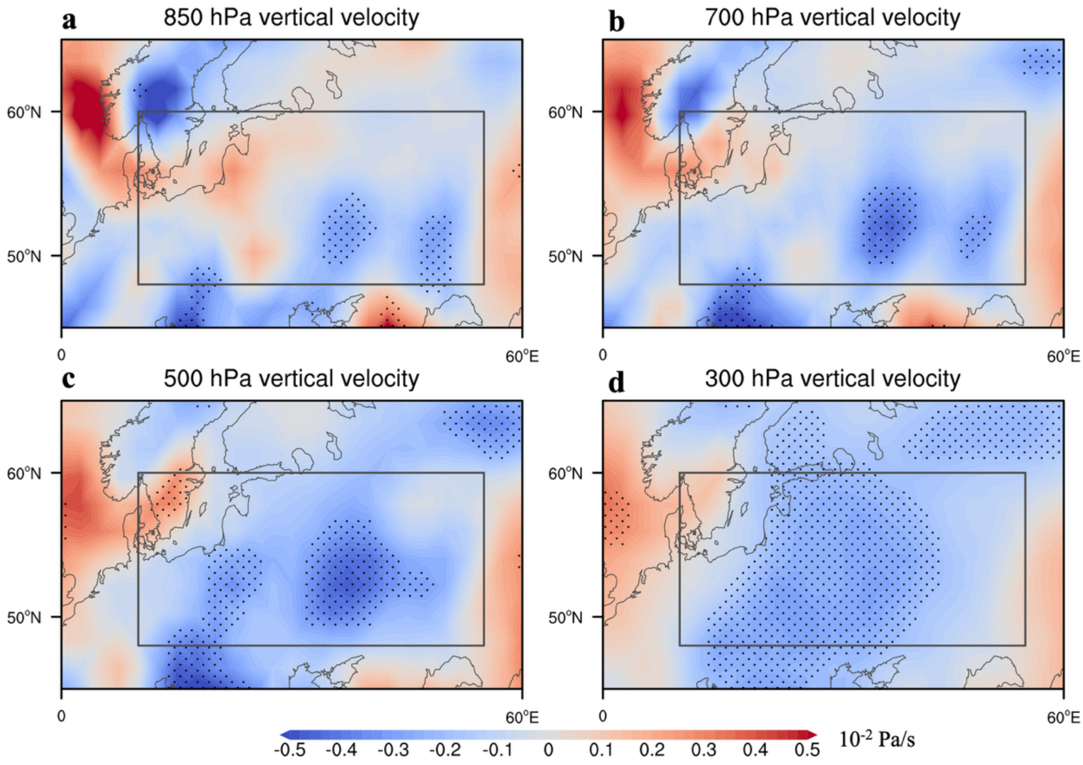

Supplementary Fig. 11: Simulated December-January average vertical velocity response. Ensemble mean responses (LessSCE minus ClimSCE) of December-January (**a**) 850 hPa, (**b**) 700 hPa, (**c**) 500 hPa, and (**d**) 300 hPa vertical velocity (10^{-2} Pa/s). Negative values correspond to upward motion. Stippling in panels (**a-d**) indicate values significant at the 90% confidence level based on the two-sided Student's t-test. The black boxes in panels (**a-d**) represent the domain of western Eurasia. The black boxes represent the domain of western Eurasia (10 °E-55 °E & 48 °N-60 °N).

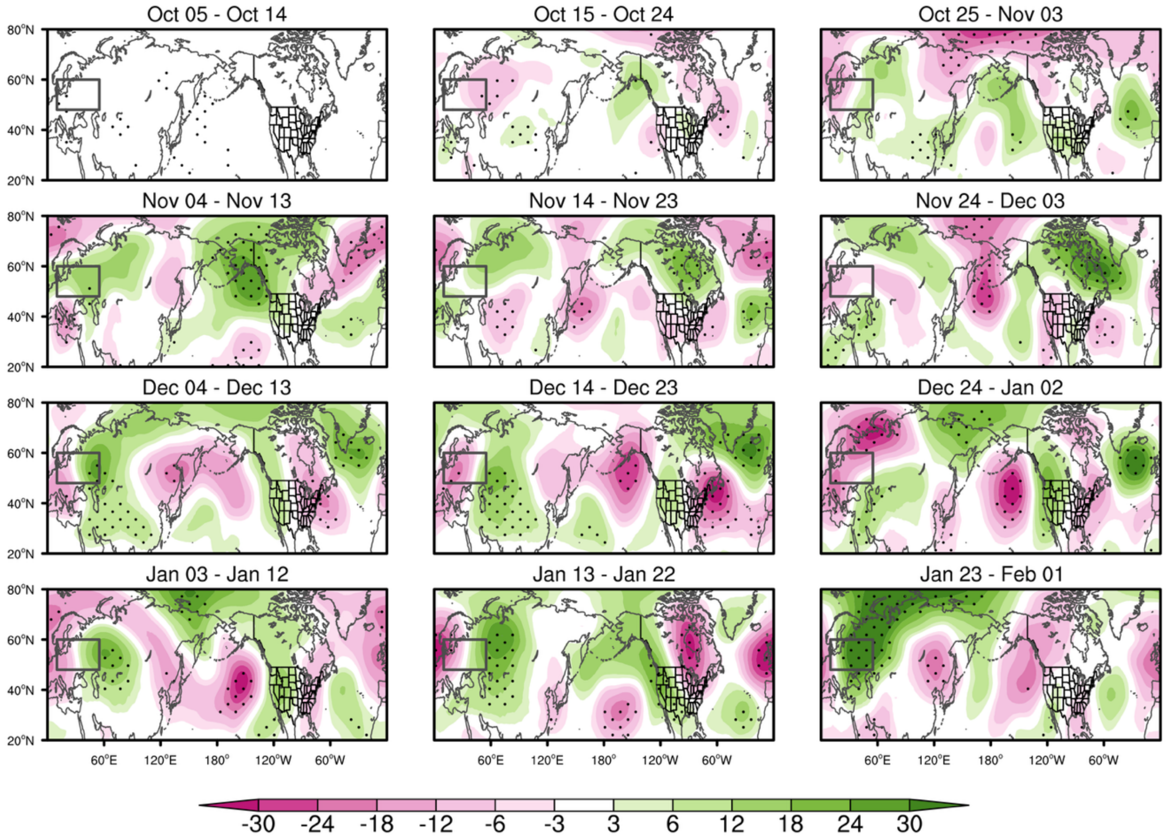

Supplementary Fig. 12: Simulated 500 hPa geopotential height responses for each 10-day stage. Each panel shows the 10-day mean geopotential height response at 500 hPa (LessSCE minus ClimSCE; shading), with black dots indicating grid points where values are statistically significant at the 90% confidence level based on the two-sided Student's t-test. Units are meters. The black boxes represent the domain of western Eurasia (10 °E-55 °E & 48 °N-60 °N).

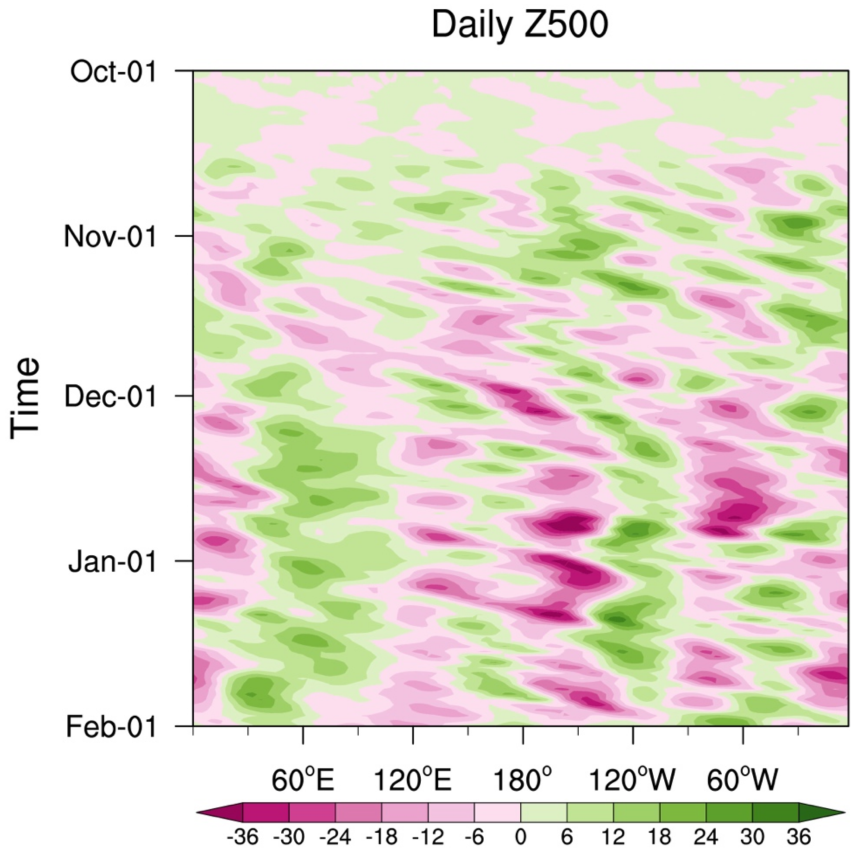

Supplementary Fig. 13: Hovmöller diagrams of daily Z500 responses averaged over 30°–50°N. This latitude band largely corresponds to the region influencing wildfire activity in Southern California. The y-axis denotes time, and the x-axis denotes longitude. Units are meters.

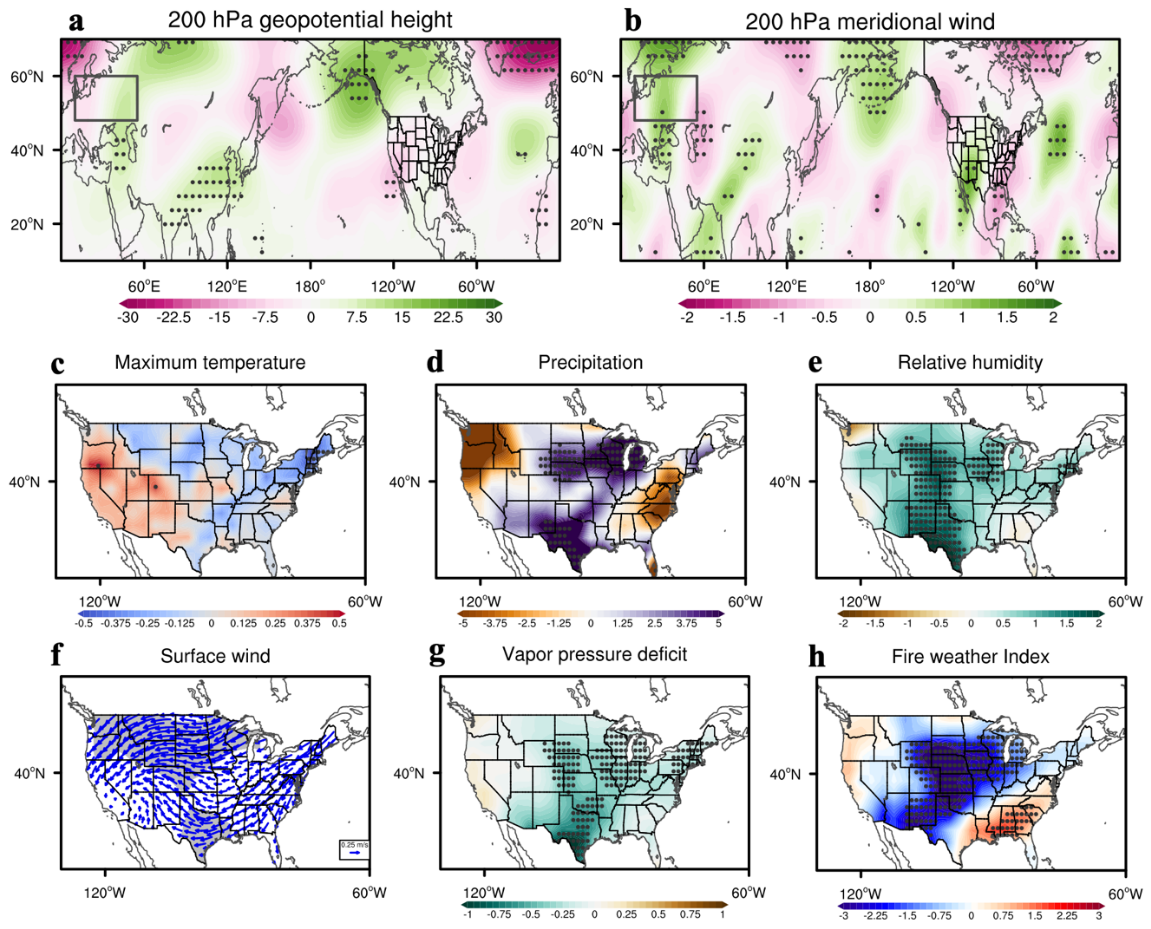

Supplementary Fig. 14: Simulated November atmospheric teleconnection and U.S. fire-weather condition response to western Eurasian snow cover extent (SCE) reduction. Ensemble mean responses (LessSCE minus ClimSCE) of November 200 hPa (**a**) geopotential height (m), (**b**) 200 hPa meridional wind (m/s), (**c**) maximum temperature (°C), (**d**) precipitation (mm/month), (**e**) relative humidity (%), (**f**) 10-m surface wind, (**g**) vapor pressure deficit (VPD, hPa), and (**h**) fire weather index (FWI, unitless). Stippling in panels (**a**-**e**) and (**g**-**h**), and shading areas in panel (**f**) indicate values significant at the 90% confidence level based on the two-sided Student's t-test. The black boxes in panels (**a**-**b**) represent the domain of western Eurasia (10 °E-55 °E & 48 °N-60 °N).

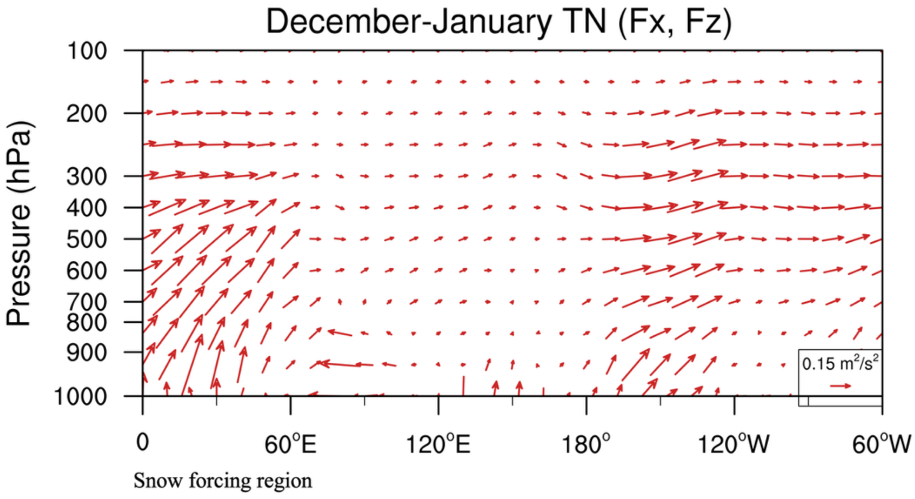

Supplementary Fig. 15: Simulated Vertical structure of wave activity flux (WAF) during December–January. A longitude–pressure cross-section of the WAF (Fx–Fz, m^2/s^2) averaged over 30 °N-60 °N along the main wave path during December–January. For visualization purposes, the vertical component of the wave-activity flux (Fz) is multiplied by a factor of 100 to account for its smaller magnitude relative to the horizontal component.

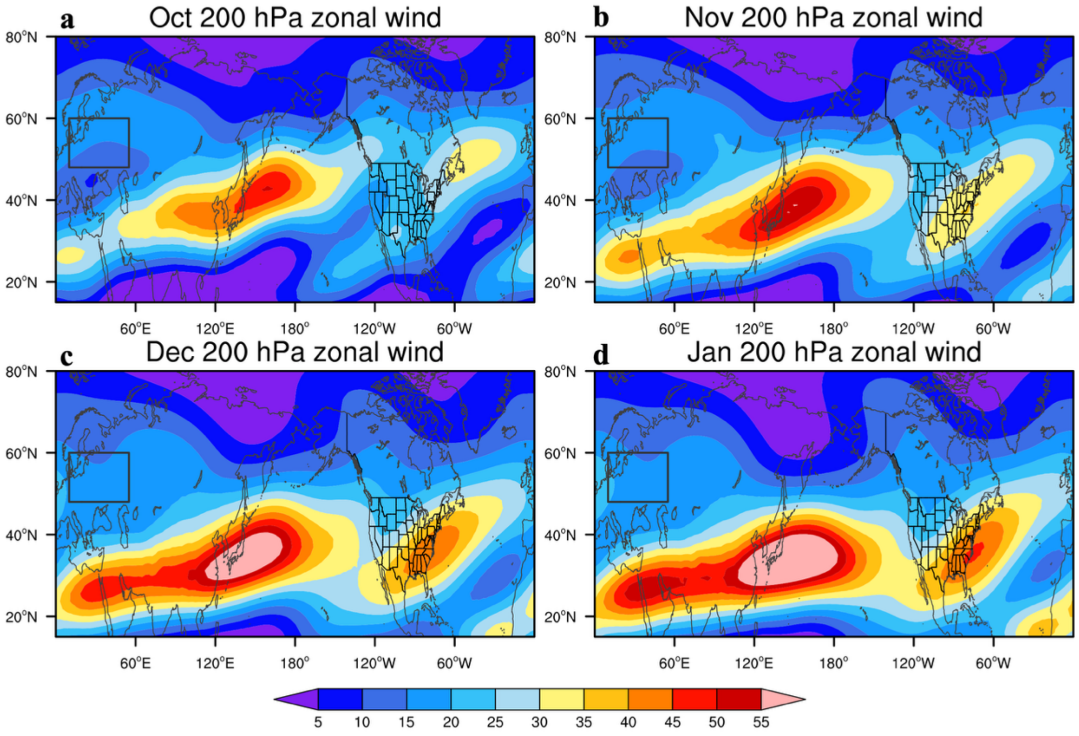

Supplementary Fig. 16: Monthly CESM2.1 long-term climatological 200 hPa zonal wind. (**a**) October, (**b**) November, (**c**) December, and (**d**) January climatological 200 hPa zonal wind in the CESM2.1 long-term control run. Units are m/s. The black boxes in panels (**a**-**d**) represent the domain of western Eurasia (10 °E-55 °E & 48 °N-60 °N).

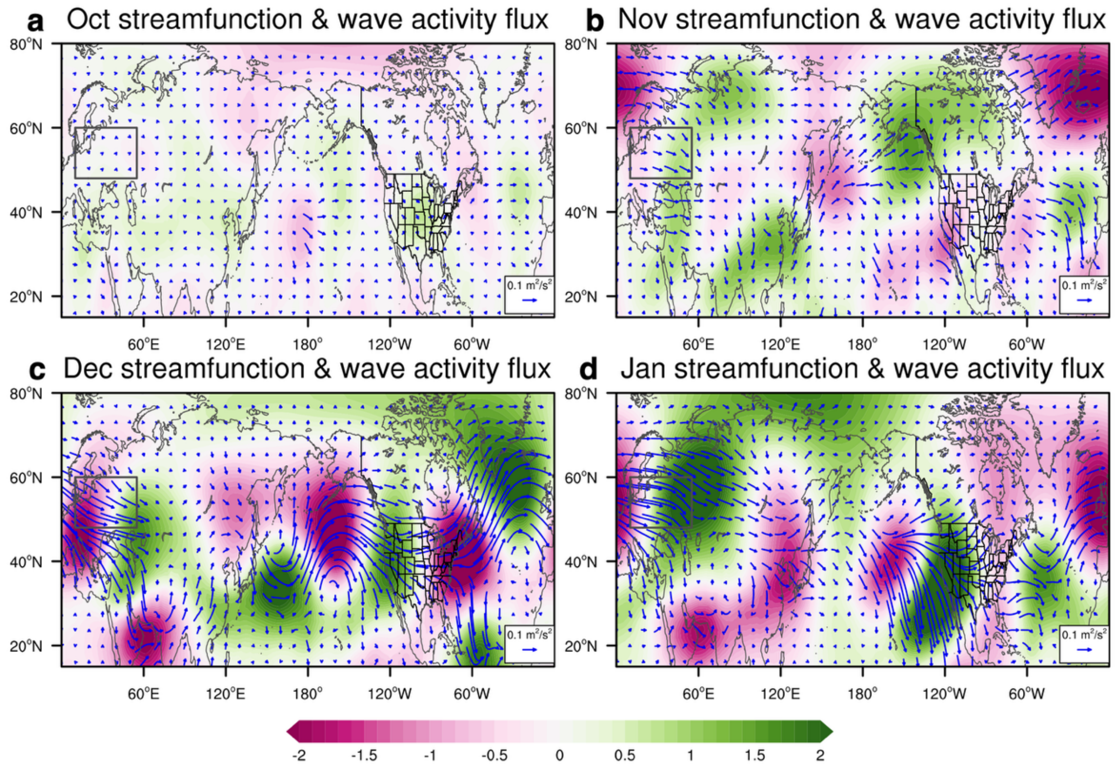

Supplementary Fig. 17: Simulated monthly streamfunction and stationary horizontal wave activity flux. Ensemble mean responses (LessSCE minus ClimSCE) of monthly 200 hPa streamfunction (shading, 10^6 m²/s) and stationary horizontal wave activity flux (WAF, vectors). The black boxes in panels (**a**)-(**b**) represent the domain of western Eurasia (10 °E-55 °E & 48 °N-60 °N).

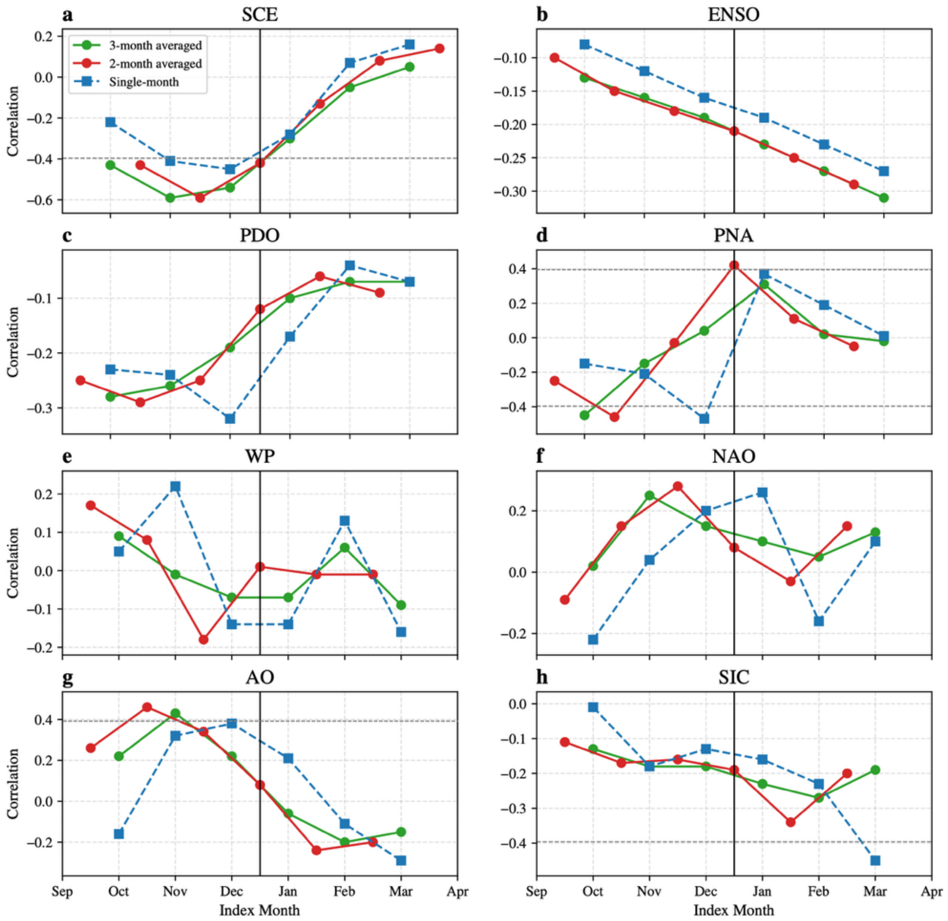

Supplementary Fig. 18: The lead-lag correlations between the Southern California December–January fire weather index (FWI) and other major climate indices. Correlation coefficients between the December–January (DJ) mean Southern California gridMET FWI and a suite of large-scale climate indices, including western Eurasian (10 °E-55 °E & 48 °N-60 °N) snow cover extent (SCE), El Niño–Southern Oscillation (ENSO), Pacific Decadal Oscillation (PDO), Pacific–North American pattern (PNA), West Pacific pattern (WP), North Atlantic Oscillation (NAO), Arctic Oscillation (AO) and North Atlantic–Canadian Arctic (210°E –300°E, 55°N –75°N) sea ice concentration (SIC) (**a**–**h**). In each panel, the green solid line shows the correlations between 3-month averaged climate index and DJ FWI; the red solid line shows the correlations between 2-month averaged climate index and DJ FWI; the blue dashed line shows the correlations between single-month climate index and DJ FWI. The horizontal axis represents the central time point of the climate index, with months to the left of the vertical black line indicating that the climate index leads the DJ mean FWI, and months to the right indicating that climate index lags the DJ mean FWI. The vertical black line marks the zero-lag condition. The horizontal dashed line denotes the 95% significance threshold based on a two-sided Student's t-test.

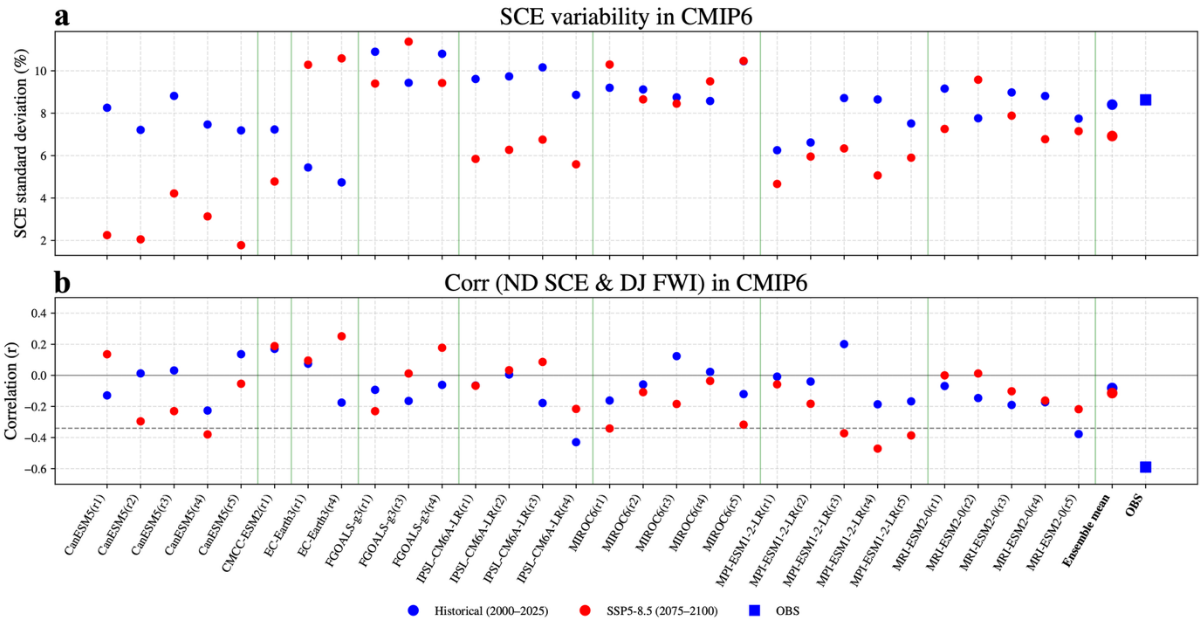

Supplementary Fig. 19: Snow cover extent (SCE) variability and SCE & fire weather index (FWI) correlations across CMIP6 ensemble members. a Standard deviation of November–December (ND) SCE in individual CMIP6 ensemble members during the historical period (2000–2025; blue dots) and under the SSP5-8.5 scenario (2075–2100; red dots). The ensemble-mean standard deviation, calculated across all 30 ensemble members, is highlighted with a larger dot. The blue square indicates the observed standard deviations of SCE from NOAA. **b** Correlation coefficients between the ND western Eurasian SCE index and the December–January (DJ) Southern California FWI index. The ensemble-mean correlation, calculated across all 30 ensemble members, is highlighted with a larger dot. The blue square indicates the observed correlation using NOAA SCE and gridMET FWI. In both panels, the dashed line marks the $p = 0.1$ significance threshold. Green vertical lines separate different ensemble members of the same model.

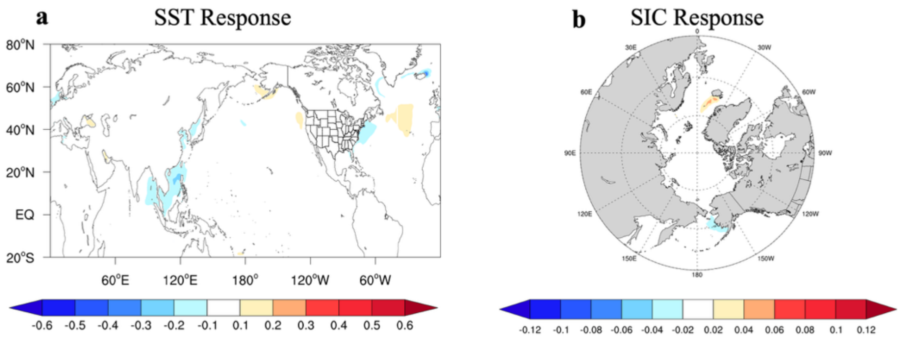

Supplementary Fig. 20: Simulated December-January oceanic responses. Ensemble mean responses (LessSCE minus ClimSCE) of December-January average (**a**) sea surface temperature (SST, °C) and (**b**) sea ice concentration (SIC, unitless).

	NOAA SCE	IMS SCE	MODIS SCE	gridMET FWI
IMS SCE	0.99 (p<0.01)			
Modis SCE	0.78 (p<0.01)	0.80 (p<0.01)		
gridMET FWI	-0.64 (p<0.01)	-0.59 (p<0.01)	-0.54 (p<0.01)	
ERA5 FWI	-0.55 (p<0.01)	-0.51 (p<0.01)	-0.45 (p<0.03)	0.97 (p<0.01)

Supplementary Table 1. Correlation coefficients between different indices. Correlation coefficients between November–December (ND) western Eurasian SCE indices from NOAA, IMS, and MODIS, and December–January (DJ) Southern California FWI indices from gridMET and ERA5, over the period 2000–2025, with El Niño–Southern Oscillation (ENSO) removed. Values in parentheses denote significance levels.

Model	Ensemble members
ACCESS-CM2	r1,r2,r3,r4,r5
ACCESS-ESM1-5	r1,r2,r3,r4,r5
CanESM5	r1,r2,r3,r4,r5
CMCC-ESM2	r1
EC-Earth3	r1,r4
FGOALS-g3	r1,r3,r4
INM-CM4-8	r1
INM-CM5-0	r1
IPSL-CM6A-LR	r1,r2,r3,r4
MIROC6	r1,r2,r3,r4,r5
MPI-ESM1-2-HR	r1,r2
MPI-ESM1-2-LR	r1,r2,r3,r4,r5
MRI-ESM2-0	r1,r2,r3,r4,r5

Supplementary Table 2. List of CMIP6 models and their corresponding ensemble members used in this study. The table includes the model names and the first five available realizations selected for the analysis.
